# LINC00431 modulates KRAS and p53 stability to drive pancreatic cancer progression under hypoxia

**DOI:** 10.1016/j.gendis.2025.101696

**Published:** 2025-05-27

**Authors:** Zhiwei Cai, Meng Liu, Weiyi Wang, Hongfei Yao, Shuo Yang, Chunjing Li, Xiao Hu, Yunlong Pu, Jianxia Ma, Chongyi Jiang

**Affiliations:** a Department of General Surgery, Pancreatobiliary Surgery Center, Huadong Hospital, Fudan University, Shanghai 200040, China; b Department of Gastroenterology, Huadong Hospital, Fudan University, Shanghai 200040, China; c Department of Laboratory Medicine, Zhongshan Hospital, Fudan University, Shanghai 200032, China

Long intergenic non-coding RNAs (lincRNAs), a subclass of long non-coding RNAs (lncRNAs) that do not overlap with other genes, play pivotal roles in cancer progression.^1^ The hypoxic tumor microenvironment, characterized by an excess of stromal cells and extracellular matrix and insufficient vascularization, is a distinctive feature of pancreatic ductal adenocarcinoma (PDAC).^2^ Nevertheless, a comprehensive screening and exploration of hypoxia-regulated lincRNAs in PDAC has not yet been conducted. This study analyzed the TCGA_PAAD dataset and identified LINC00431 as a hypoxia-responsive lincRNA. LINC00431 functions as an oncogenic lincRNA by modulating the protein levels of p53 and KRAS through the E3 ligase TRAF7 (tumor necrosis factor receptor-associated factor 7) in PDAC. These findings elucidate a novel mechanism in PDAC progression mediated by LINC00431, proposing it as a potential target for therapeutic intervention in PDAC.

To identify hypoxia-regulated oncogenic lincRNAs in PDAC progression, we developed a bioinformatic screening procedure (Fig. S1A). Using the TCGA_PAAD dataset, we identified eight significantly up-regulated and hypoxia-enriched lincRNAs in PDAC patients (Fig. 1A; Fig. S1B–E) and further filtered them by prognosis relevance (Fig. S1F), selecting four lincRNAs (Fig. 1A, B). Among these, only LINC00431 (Fig. 1B) had not been previously reported, leading us to focus on it. Coding-Potential Assessment Tool (CPAT) analysis confirmed the non-coding nature of LINC00431 (Fig. S1G).

**Figure 1:**
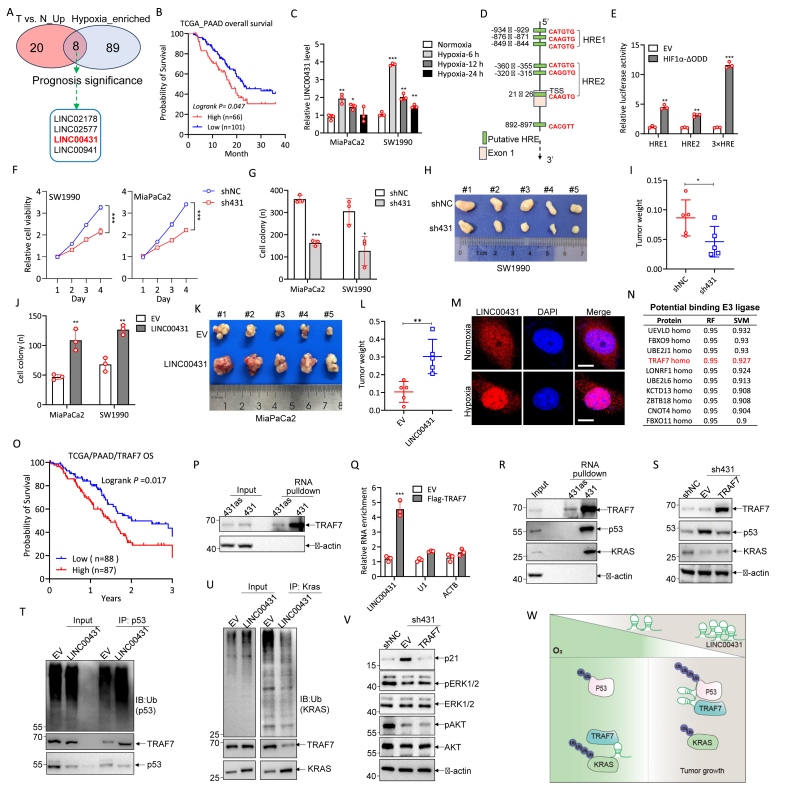
LINC00431 promotes pancreatic ductal adenocarcinoma (PDAC) progression under hypoxia. **(A)** The Venn diagram showing the common lincRNAs: significantly up-regulated lincRNAs in PDAC tumors versus adjacent tissues (*n* = 4) (log_2_ fold change ≥ 1, *p* ≤ 0.05, shown in brick red), and lincRNAs significantly enriched in the top 4 hypoxia score PDAC tumors compared with the lowest 4 hypoxia score PDAC tumors (log_2_ fold change ≥ 1, *p* ≤ 0.05, shown in light blue). The raw data were obtained from the TCGA portal. ∗*p* < 0.05. **(B)** Prognosis analysis of LINC00431 in TCGA_PAAD patients. **(C)** Quantitative PCR analysis of LINC00431 expression in MiaPACA2 and SW1990 cells under hypoxic treatment. ∗∗∗*p* < 0.0001. **(D)** The schematic diagram depicting the location of the potential hypoxia-responsive elements (HREs) in the LINC00431 promoter region (−1000 nt ∼ +1000 nt). **(E)** Dual-luciferase reporter experiments revealed that LINC004331 was regulated by HIF1α (ΔODD, ODD domains deletion) in HEK-293T under normoxia. ∗∗*p* < 0.001 and ∗∗∗*p* < 0.0001. **(F)** CCK8 cell viability assays revealed that depletion of LINC00431 inhibited SW1990 and MiaPaCa2 cell proliferation. ∗∗∗*p* < 0.0001. **(G)** Cellular colony formation assays indicated that knockdown of LINC00431 inhibited MiaPaCa2 and SW1990 cell proliferation and growth. ∗*p* < 0.05 and ∗∗∗*p* < 0.0001. **(H, I)** Knockdown of LINC00431 inhibited the growth of SW1990 cells in subcutaneous tumor models. Tumor size (H) and weight (I) were shown. ∗*p* < 0.05. **(J)** Cellular colony formation assays indicated that overexpression of LINC00431 promoted MiaPaCa2 and SW1990 cell proliferation and growth. ∗∗*p* < 0.01. **(K, L)** Overexpression of LINC00431 promoted the growth of MiaPaCa2 cells in subcutaneous tumor models. Tumor size (K) and weight (L) were shown. ∗∗*p* < 0.01. **(M)** RNA-fluorescence *in situ* hybridization analysis of the subcellular localization of LINC00431 in SW1990 cells under normoxia and hypoxia. **(N)** Top 10 potential binding partners of LINC00431, data predicted by the RPISeq online tool. **(O)** Overall survival analysis of TRAF7 in the TCGA_PAAD dataset. **(P)** RNA pulldown assays revealed that LINC00431 bound to TRAF7 in SW1990 cells pre-treated under hypoxia for 12 h. **(Q)** Quantitative PCR analysis of indicated RNAs in SW1990 RNA-immunoprecipitation samples. Cells were treated under hypoxia for 12 h ∗∗∗*p* < 0.0001. **(R)** RNA pulldown assays revealed that p53 and KRAS bound to LINC00431 in SW1990 cells pre-treated under hypoxia for 12 h. **(S)** Immunoblotting analysis of TRAF7, p53, and KRAS proteins in the indicated SW1990 stable cell lines under hypoxia for 12 h. **(T)** Immunoprecipitation of p53 in SW1990 cells with or without LINC00431 overexpression under hypoxia for 12 h. The cells were treated with MG132 (1 μg/mL) in the last 2 h. Ubiquitin (Ub), TRAF7, and p53 were immunoblotted. **(U)** Immunoprecipitation of KRAS in SW1990 cells with or without LINC00431 overexpression under hypoxia for 12 h. The cells were treated with MG132 (1 μg/mL) in the last 2 h. Ubiquitin (Ub), TRAF7, and KRAS were immunoblotted. **(V)** Immunoblotting analysis of p21, pERK1/2, and pAKT (s473) was performed on the indicated SW1990 stably transfected cells, which were cultured under hypoxia for 12 h. **(W)** The proposed working model of LINC00431 in PDAC: Under normoxia, LINC00431 expression is low and guides TRAF7 to KRAS, promoting KRAS ubiquitination. Upon hypoxia, LINC00431 expression is elevated and tends to guide TRAF7 to bind to p53, promoting p53 ubiquitination.

We then confirmed that LINC00431 was a hypoxia-responsive lncRNA by culturing PDAC cell lines MiaPaCa2 and SW1990 under normoxia or hypoxia for durations of 6, 12, and 24 h (Fig. 1C; Fig. S1H). Since hypoxia-inducible factor-1 alpha (HIF1α) is the most critical transcription factor under hypoxia,^2^ we verified whether LINC00431 was regulated by HIF1α. Overexpression of the oxygen-dependent degradation domain (ODD) deleted HIF1α (HIF1α-ΔODD) enhanced the expression of LINC00431 (Fig. S1I, J). Moreover, HIF1α knockdown reduced the expression of LINC00431 in both MiaPaCa2 and SW1990 cells (Fig. S1K, L). Furthermore, in the promoter region (−1000 nt to +1000 nt) of LINC00431 (Fig. 1D), seven hypoxia-responsive elements (HREs) were identified, suggesting that LINC00431 may be a direct target of HIF1α. The dual-luciferase reporter assays revealed that HIF1α might bind to both HRE1 and HRE2 in SW1990 cells (Fig. 1E). In addition, chromatin immunoprecipitation-sequencing data from the ENCODE database revealed that HIF1α was capable of binding to the promoter region of LINC00431 (Fig. S1M). Altogether, all these results suggest that LINC00431 is a direct target of HIF1α under hypoxia.

To explore LINC00431's biological roles, we performed Gene Set Enrichment Analysis (GSEA) using TCGA-PAAD RNA sequencing data, revealing positive correlations with HALLMARK_E2F_targets (Fig. S2A), HALLMARK_MYC_targets (Fig. S2B), and HALLMARK_Hypoxia (Fig. S2C). These findings suggest that LINC00431 may act as an oncogene by regulating these pathways in PDAC. Stable knockdown MiaPaCa2 and SW1990 cells were constructed, with sh#1 selected for further study (Fig. S2D). CCK8 viability (Fig. 1F) and colony formation assays (Fig. 1G) showed that LINC00431 silencing inhibited PDAC cell proliferation and growth, confirmed by subcutaneous tumor assays *in vivo* (Fig. 1H, I). Conversely, LINC00431 overexpression promoted cell proliferation and growth *in vitro* and *in vivo* (Fig. 1J–L). Additionally, silencing LINC00431 reduced cyclin B1 and cyclin-dependent kinase 4 (CDK4) protein levels (Fig. S2E), and these effects were reversed by overexpression (Fig. S2F). These results indicate that hypoxia-induced LINC00431 promotes PDAC proliferation and growth *in vitro* and *in vivo*.

To explore the biological role of LINC00431, we performed cyto-nuclear fractionation (Fig. S2G) and RNA-fluorescence *in situ* hybridization (Fig. 1M). Results showed that LINC00431 was localized in both the cytoplasm and the nucleus. Hypoxia increased its nuclear accumulation in SW1990 cells (Fig. 1M; Fig. S2G), possibly due to reduced nuclear RNA export factor 1 (NXF1) expression (S2G).

Using the RPISeq online website tool, we identified several ubiquitin E2 and E3 ligases as potential LINC00431 binding partners (Fig. 1N). TCGA_PAAD analysis showed that E3 ligase TRAF7 was significantly elevated in PDAC tissues (Fig. S2H) and predicted poor prognosis (Fig. 1O). GSEA further revealed that TRAF7 positively correlated with MYC- (Fig. S2I) and E2F- (Fig. S2J) associated gene sets, consistent with LINC00431 (Fig. S2A, B). Interestingly, TRAF7 negatively correlated with the KRAS_signaling_up gene set (Fig. S2K), suggesting it may inhibit KRAS signaling in PDAC.

We then sought to investigate the interaction between LINC0431 and TRAF7. Intriguingly, RNA pulldown assays suggest that LINC00431 could bind to TRAF7 (Fig. S2L; Fig. 1P), and RNA immunoprecipitation assays, on the other hand, revealed that TRAF7 could immunoprecipitate LINC00431 (Fig. S2M; Fig. 1Q). TRAF7 has been reported to mediate the degradation of tumor suppressor p53 and oncogene KRAS.^3^^,^^4^ Consistently, we confirmed its interaction with both p53 and KRAS (Fig. S2N).

These results led us to investigate whether LINC00431 modulated the protein levels of p53 and KRAS in PDAC cells through TRAF7. Using SW1990 cells, which harbor the KRAS^G12D^ mutation, we firstly discovered that LINC00431 could pull down both p53 and KRAS proteins, including wild-type KRAS and the KRAS^G12D/G12C^ mutants (Fig. 1R; Fig. S2O). Secondly, knocking down LINC00431 significantly increased the protein level of p53 and inhibited KRAS protein under hypoxia conditions (Fig. 1S). However, TRAF7 overexpression restored p53 protein levels by increasing its binding to p53 (Fig. 1S; Fig. S2P). In contrast, KRAS protein levels remained unchanged in SW1990 cells (Fig. 1S), while the mRNA stability of both p53 and KRAS was unaffected (Fig. S2Q).

Thirdly, LINC00431 overexpression enhanced the interaction between TRAF7 and p53, leading to increased ubiquitination of p53 (Fig. 1T). Lastly, LINC00431 overexpression reduced the interaction between TRAF7 and KRAS, thereby inhibiting the ubiquitination of KRAS protein (Fig. 1U). These findings suggest that LINC00431 could regulate the ubiquitination levels of p53 and KRAS under hypoxia by modulating their interactions with TRAF7. We also found that TRAF7 overexpression enhanced the expression of the p53 target protein p21 (Fig. 1V) and other downstream targets (Fig. S2R) under hypoxia, which were activated by LINC00431 knockdown. However, both extracellular signal-regulated kinase (ERK)1/2 and protein kinase B (Akt) signaling pathways, which are canonical downstream signaling of KRAS, could not be restored by TRAF7 overexpression in LINC00431 knockdown cells (Fig. 1V). It appears that ERK1/2 and Akt signaling are reduced to baseline levels upon LINC00431 knockdown, and TRAF7 overexpression is unable to restore them. Functionally, TRAF7 overexpression in SW1990-sh431 cells mitigated the inhibition of cell proliferation caused by LINC00431 silencing (Fig. S2T).

Based on our findings, we propose that under hypoxic conditions, LINC00431 accumulates in the nucleus and redirects TRAF7 from cytoplasmic KRAS to nuclear p53, leading to p53 degradation and KRAS stabilization. This shift facilitates PDAC progression. These results also suggest that targeting LINC00431 with antisense oligonucleotides, in combination with KRAS inhibitors (*e.g.*, AMG 510) or p53 activators (*e.g.*, APR-246), may provide a promising therapeutic strategy for PDAC (Fig. 1W).

## CRediT authorship contribution statement

**Zhiwei Cai:** Writing – original draft, Project administration, Methodology, Investigation, Formal analysis, Data curation, Conceptualization. **Meng Liu:** Methodology, Investigation, Data curation. **Weiyi Wang:** Software, Methodology, Investigation. **Hongfei Yao:** Methodology, Investigation. **Shuo Yang:** Methodology, Investigation. **Chunjing Li:** Investigation. **Xiao Hu:** Investigation. **Yunlong Pu:** Investigation. **Jianxia Ma:** Supervision, Conceptualization. **Chongyi Jiang:** Writing – review & editing, Supervision, Funding acquisition, Conceptualization.

## Ethics declaration

All animal procedures were conducted in strict accordance with the ARRIVE guidelines and complied with the U.K. Animals (Scientific Procedures) Act, 1986, as well as other relevant ethical guidelines. Approval for the experimental protocol was granted by the Ethics Committee of Huadong Hospital, ensuring adherence to all regulatory requirements for animal care and use.

## Funding

This research was supported by the 10.13039/501100001809National Natural Science Foundation of China (No. 82372968), the 10.13039/501100014137Shanghai Shenkang Hospital Development Center (No. SHDC2024CRI080), the Medical Engineering Joint Fund of Fudan University, Shanghai, China (No. XM03231533), and the Shanghai Science and Technology Development Funds (Shanghai, China) (No. 22YF1406200).

## Conflict of interests

The authors declared no conflict of interests.
