# Supplementary material for: LINC00431 modulates KRAS and p53 stability to drive pancreatic cancer progression under hypoxia
Source: Genes Dis. 2025 May 27;13(1):101696. doi: 10.1016/j.gendis.2025.101696 (PMC12466122; doi:10.1016/j.gendis.2025.101696)
Supplement: Multimedia component 1 [file mmc1.docx]

**Supplementary data for**

**LINC00431 Modulates KRAS and p53 Stability to Drive Pancreatic Cancer Progression Under Hypoxia**

Zhiwei Cai^1, #^, Meng Liu^1, #^, Weiyi Wang^1, #^, Hongfei Yao^1,#^, Chunjing Li^1^, Xiao Hu^1^, Yunlong Pu^1^, Jianxia Ma^2, *^, Chongyi Jiang^1,*^

^#^ These authors contributed equally to this work.

^*^ Correspond to Prof. Chongyi Jiang, E-mail: jiangzhongyi9@sina.com, and Dr. Jianxia Ma, E-mail: yz_mjx@fudan.edu.cn

This file includes:

Supplementary Fig. S1 to S2

Methods and Materials


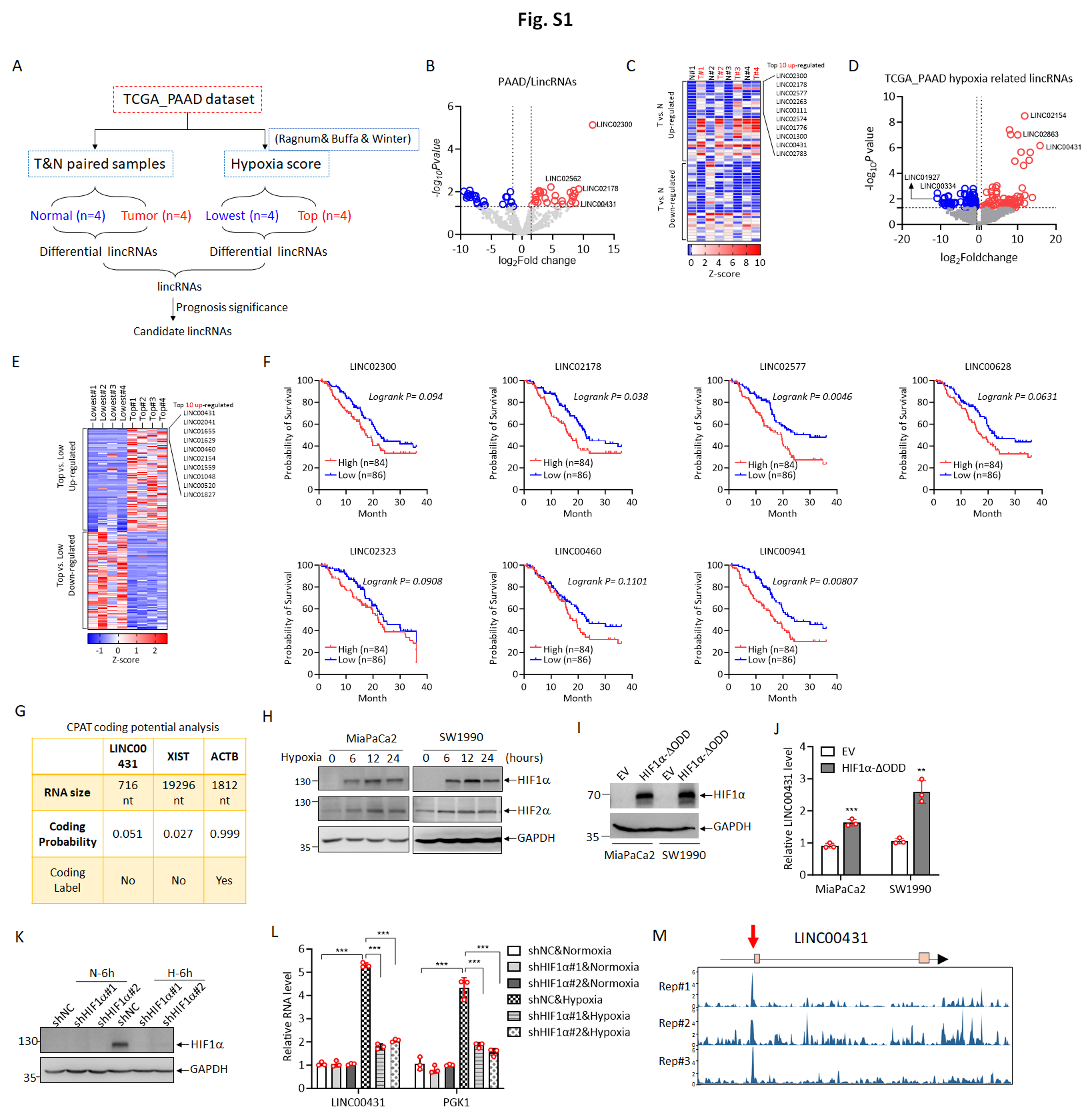


Figure S1. LINC00431 is regulated by hypoxia in PDAC. (A) Schematic diagram illustrating the bioinformatic screening of hypoxia-regulated lncRNAs within the TCGA_PAAD dataset. (B, C) Volcano plot (B) and heatmap (C) illustrating the differential lincRNAs (Tumor vs. Normal, log2FC ≥ 1, P ≤ 0.05) in PDAC patients. For (B) Red and Blue cycles indicate Up- and Down-regulated lincRNAs in PDAC patients, respectively. (D, E) Volcano plot (D) and heatmap (E) illustrating the differential lincRNAs (Hypoxia score high vs. Hypoxia low, log2FC ≥ 1, P ≤ 0.05) in PDAC patients. For (D) Red and Blue cycles indicate Up- and Down-regulated lincRNAs in PDAC patients, respectively. (F) Overall survival analysis of indicated lincRNAs in PDAC patients, data from TCGA_PAAD dataset. (G) Coding potential analysis of LINC00431. XIST was used as noncoding RNA control, and ACTB as coding control. (H) Immunoblotting analysis of HIF1α and HIF2α in hypoxia treated MiaPACA2 and SW1990 cells. (I) Immunoblotting analysis of HIF1α in MiaPACA2 and SW1990 cells with or without overexpression of HIF1α-ΔOOD (ODD domain deleted HIF1α mutant). (J) qPCR analysis of LINC00431 expression in MiaPACA2 and SW1990 cells with or without overexpression of HIF1α-ΔOOD. ** indicates P < 0.001, *** indicates P < 0.0001. (K) Immunoblotting analysis of HIF1α in MiaPaCa2 cells with or without knockdown of HIF1α under normoxia and hypoxia for 6 hours. N, Normoxia; H, Hypoxia. (L) qPCR analysis of LINC00431 and PGK1 mRNA in MiaPACA2 with or without knockdown of HIF1α under normoxia and hypoxia. *** indicates P < 0.0001. (M) ChIP-seq data revealed that HIF1 binds to the promoter region of LINC00431, data from ENCODE. Red arrow indicates the TSS (Transcription start site).


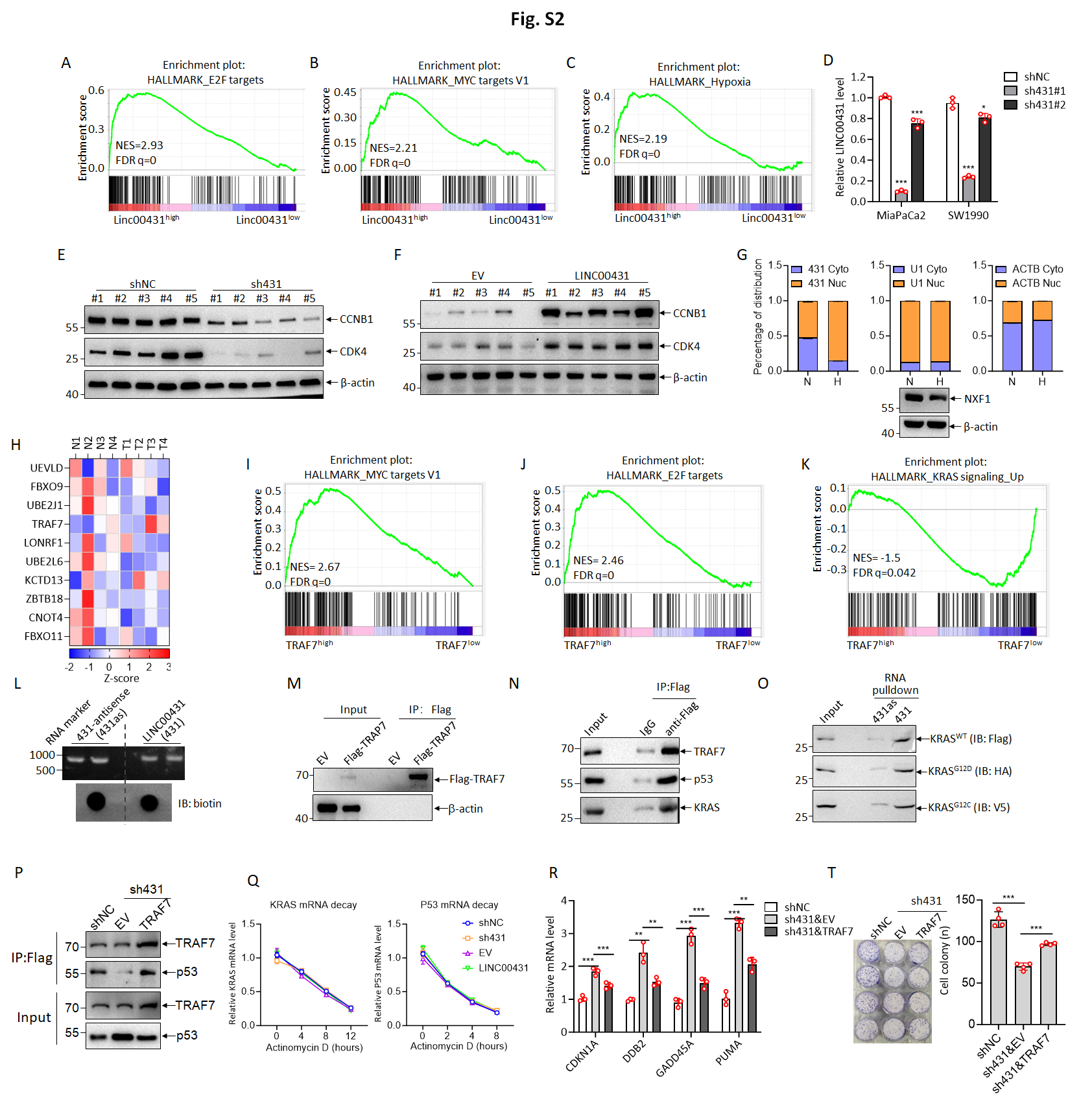


Figure S2. LINC00431 promotes PDAC cell proliferation and growth through TRAF7. (A-C) signle gene geneset enrichment analysis (sgGSEA) revealed that LINC00431 positively correlated to E2F (A), MYC (B) and Hypoxia (C) related genesets. (D) qRT-PCR analysis of the knockdown efficiency of sh431#1 and sh431#2 in MiaPaCa2 and SW1990 cells. (E) Immunoblotting analysis of cyclin B1 and CKD4 proteins in SW1990-shNC and -sh431 tumors. (F) Immunoblotting analysis of cyclin B1 and CKD4 proteins in MiaPaCa2-EV and -LINC00431 tumors. (G) qRT-PCR analysis of the subcellular distribution of LINC00431 in SW1990 cells under hypoxia and normoxia (Top). U1 snRNA as nucleus marker, ACTB mRNA as cytoplasm marker. Nuc, nucleus; Cyto, cytoplasm; N, normoxia; H, hypoxia. Immunoblotting analysis of NXF1 protein level in SW1990 cells under normoxia and hypoxia (bottom). (H) Heatmap depicting the expression of indicated E3 ligases in TCGA_PAAD dataset. (I-K) sgGSEA analysis revealed that TRAF7 is positively associated with genesets related to MYC (I) and E2F (J) and negatively correlated with the KRAS signaling up-regulated geneset (K). (L) In vitro transcription of biotin-labelled LINC00431 and its antisense (Top) and immunoblotting analysis of biotin labeling efficiency (bottom). (M) anti-Flag immunoprecipitation in SW1990 cells after transfected with empty vector (EV, flag only) and Flag-tagged TRAF7. (N) Immunoprecipitation analysis of TRAF7 and p53 or KRAS under hypoxia in SW1990 cells. (O) RNA-pulldown revealed that LINC00431 binds to both wild type KRAS (KRAS^WT^) and mutated KRAS (KRAS^G12D/G12C^) in SW1990 cells, cells were pre-treated under hypoxia for 12 hours. (P) Immunoprecipitation analysis of TRAF7-Flag and p53 in indicated cells. (Q) RNA decay analysis of P53 and KRAS mRNA in indicated cells, Actinomycin D 5 μg/mL. (R) qPCR analysis of indicated p53 targets in SW1990 indicated samples. ** indicates P < 0.01, *** indicates P < 0.0001. (T) Cellular colony formation assays showed that overexpression of TRAF7 reverses the inhibition of cell proliferation in SW1990-sh431 cells. Representative images (left) and statistical analysis (right) are presented. *** indicates P < 0.0001.

**Materials and Methods**

Cell Culture

The pancreatic ductal adenocarcinoma (PDAC) cell lines, MiaPaCa-2 and SW1990, were acquired from the American Type Culture Collection (ATCC) in Manassas, VA. Additionally, the HEK-293T cell line was sourced from Shanghai Zhuyi Biotechnology. These cell lines were cultured in either DMEM or RPMI-1640 medium, each supplemented with 10% fetal bovine serum (FBS) and maintained at standard conditions of 37°C with 5% CO2. For hypoxic experiments, cells were cultured in a hypoxic incubator (Thermo fisher) set to an oxygen concentration of 1%, and treatments were conducted over specified durations. For RNA decay analysis, SW1990 related cells were treated with 5 μg/mL Actinomycin D for indicated times.

Plasmids and stable cell constructions

We acquired plasmids for overexpression, including those for LINC00431, TRAF7, and an empty vector (EV, pLVX-puro), as well as shRNA vectors targeting LINC00431 (sh #1, sh#2), and a negative control (NC, pLKO.1-puro&EGFP), all from Shanghai Zhuyi Biotech, Shanghai, China. Lentiviruses were produced in HEK-293T cells using pMDG and ∆8.9 as the lentiviral backbone. Lentiviral supernatants were harvested 24 and 48 hours after transfection and used to infect the PDAC cell lines MiaPaCa2 and SW1990. Selection of positively transduced cells was achieved using 2 µg/mL puromycin, 48 hours post-infection.

RNA Isolation and Quantitative PCR (qPCR)

RNA was extracted from selected cell samples using TRIzol reagent (Takara, China) according to the manufacturer's protocol. For reverse transcription, 2 µg of total RNA was converted into cDNA using HiScript Reverse Transcriptase (Vazyme, China). Quantitative real-time PCR (qRT-PCR) was then performed using the ABI-Q5 real-time RT-PCR system and SYBR Green Real-time PCR Master Mix (Vazyme, China). 18S RNA was used as the endogenous reference. The sequences of the primers used are listed in Table S1.

Western blot

For experiments, cells were seeded at a density of 5×10^5^ per 6-cm dish and allowed 24 hours to adhere. Cells were then lysed using either 1% SDS or the specified lysis buffer, followed by boiling and separation via SDS-PAGE. Proteins were transferred to nitrocellulose membranes which were blocked for one hour with 5% skim milk at room temperature. Overnight incubation at 4°C was performed with primary antibodies: HIF1α, HIF2α, and CCNB1 from Cell Signaling Technology (CST, 1:1000 each), CDK1, CCNB1, TRAF7, p53, β-actin, and GAPDH from Proteintech. The following day, after washing with PBST (0.05% Tween-20), membranes were incubated with the appropriate HRP-conjugated secondary antibodies, either anti-mouse or anti-rabbit IgG-HRP.

CCK8 assay for cell viability

PDAC cells were seeded at a density of 5×10^4^ cells per well in 96-well plates and cultured for both 1 and 2 days. After this period, the culture medium was discarded, and the cells were washed once with PBS. Subsequently, CCK-8 reagent was added to each well at a 1:10 ratio and the cells were incubated at 37°C in the dark for 30 minutes. Absorbance was measured at 450 nm to quantitatively assess cell viability.

Cell colony formation assay

The designated population of 1000 cells in 3 mL were seeded in 6-well plates and incubated at 37°C with 5% CO_2_ for 10-14 days, with medium changes every 2-3 days. After this period, the colonies were fixed and stained with crystal violet. The number of colonies per well was then documented and photographed. This method facilitated the assessment of the clonogenic potential of the cells.

Cell cycle analysis

PDAC cells from various experimental groups were seeded in 6-well plates and cultured for 24 hours before being harvested. After washing with PBS, the cells were fixed with 70% ethanol. Subsequently, RNase A was added to treat the RNA, and the DNA was stained using propidium iodide (PI) dye. Flow cytometry was then utilized to analyze the cell cycle, collecting data to quantify the distribution of cells across different phases. Data analysis was performed using FlowJo software, resulting in a detailed cell cycle distribution chart. This comprehensive method provides a thorough examination of how manipulations, such as alterations in LINC00431, impact cell cycle dynamics in PDAC cells.

Subcellular fractionation

PDAC cells (1×10^7^) were processed using NE-PER Nuclear and Cytoplasmic Extraction Reagents (Thermo, USA) following the manufacturer’s instructions. The resulting cytoplasmic lysates and nuclear pellets were separately treated with 1 mL of Trizol for RNA extraction and 0.2–0.5 mL of 2×SDS for protein extraction, respectively. Subsequently, RNA from an equivalent number of cells was converted into cDNA using the protocol provided for qPCR, with specific primers listed in Table S1. The isolated proteins were then analyzed by western blotting to assess protein expression levels.

Fluorescence in situ hybridization (FISH)

PDAC cells were first fixed and permeabilized to enhance probe penetration. Custom-designed fluorescent probes, targeting specific RNA sequences, were then applied to the cells. Following the hybridization period, excess probes were washed away. Subsequently, the cells were counterstained with fluorescent dyes to aid in visualization. Examination under a fluorescence microscope allowed for the detection of labeled probes, which emitted distinct signals, facilitating the precise localization and quantification of the targeted RNA molecules within the cellular environment.

RNA immunoprecipitation (RNA-IP) assay

The RNA-IP assay was conducted using the RIP assay reagent kit from Millipore, USA, following the manufacturer's protocol. Initially, 5×10^7^ SW1990 cells were cultured under hypoxia for 12 hours and then lysed by using RIP buffer. Subsequently, 5 µg of anti-Flag antibody (Sigma) and 5 µg of control mouse IgG (Sigma) were incubated with the cell lysates overnight at 4°C. Protein A/G beads (Thermo, USA) were then used to capture the antibody complexes. After the incubation, RNA bound to the antibody-bead complexes was purified for quantitative PCR (qPCR) analysis. Concurrently, the precipitated proteins were processed for western blotting to assess the specificity and efficiency of the immunoprecipitation.

RNA pulldown

5 × 10⁷ SW1990 cells were cultured under hypoxic conditions for 12 hours and then used for RNA pull-down assays with biotin-labeled RNA, following a series of procedures^5^. RNA pull-down assays were performed by initially synthesizing LINC00431 and its antisense RNA using an RNA transcription kit (ROCHE) with biotin-UTP incorporation, followed by purification using TRIzol reagent (Vazyme). The transcribed RNAs were then structured by incubating in RNA structure buffer (10 mM Tris-HCl, pH 7.0, 0.1 M KCl, 10 mM MgCl2) and heating to 72°C for 2 minutes. These RNAs were subsequently incubated with PDAC cell lysates at 4°C for four hours, followed by an hour's incubation with Streptavidin beads (Thermo, USA) at room temperature to allow for the formation of RNA-protein complexes. After five washing cycles, the complexes were analyzed using western blotting to identify and characterize the interacting proteins.

Subcutaneous tumor formation assays

BALB/c female nude mice, supplied by Shanghai Linchang Biotech, were subcutaneously injected with 2×10^6^ specified stable cells on both flanks. After a 24-day observation period, the mice were euthanized, and the tumors were excised, visually documented, and weighed. All animal procedures were conducted in strict accordance with the ARRIVE guidelines and complied with the U.K. Animals (Scientific Procedures) Act, 1986, as well as other relevant ethical guidelines. Approval for the experimental protocol was granted by the Ethics Committee of Huadong hospital, ensuring adherence to all regulatory requirements for animal care and use.

Dual-luciferase reporter assay

Initially, plasmids containing hypoxia-responsive elements, HRE1 and HRE2, were engineered into the pLG3 vector and these vectors were purchased from Shanghai Zhuyi biotech. These plasmids were then co-transfected with HIF-1α and LINC00431 into 293T cells. Cell samples were harvested 12 hours post-transfection to evaluate the direct interaction between HIF-1α and LINC00431. The assessment was conducted using a dual-luciferase reporter assay, where luciferase activity indicated HIF-1α function and Renilla luciferase activity served as a measure of LINC00431 expression. The results were quantified by calculating the ratio of luciferase to Renilla luciferase activities, providing a quantifiable metric to confirm the direct interaction between HIF1α and LINC00431.

Statistical Analysis

Data are presented as means ± SEM. Statistical comparisons between groups were performed using multiple comparison tests with Bonferroni-Dunn correction for multiple testing adjustments. A p-value of less than 0.05 was considered statistically significant. To ensure rigorous validation and enhance the reliability of our findings, each experiment was independently repeated at least three times.

Table S1 Sequences of primers used in this study.

| Name | Sequence (5’-3’) |
| --- | --- |
| LINC00431 primer | F: GCACACGTCGGAGTTGAATG |
|  | R: ACCCTTGCTGTAGATTTTGCTA |
| PGK1 primer | F: CGGGGAACCAAAGCTCTCAT |
|  | R: ATTGCTGAGAGCATCCACCC |
| CDK1 primer | F: CTTGGCTTCAAAGCTGGCTC |
|  | R: CATGGCTACCACTTGACCTGT |
| CDK4 primer | F: CAGTGTACAAGGCCCGTGAT |
|  | R: CAGTCGCCTCAGTAAAGCCA |
| CCNB1 primer | F: AACATCTGGATGTGCCCCTG |
|  | R: GGTCTCCTGCAACAACCTGA |
| ACTB primer | F: AAGGATTCCTATGTGGGCGAC |
|  | R: CGTACAGGGATAGCACAGCC |
| U1 primer | F: AAGAGTGAGGCGTATGAGGC |
|  | R: CGCCAACCAAGACACAATCC |
| 18S RNA | F: GTAACCCGTTGAACCCCATT |
|  | R: CCATCCAATCGGTAGTAGCG |
